# Patient journey and resources mapping to implement a praziquantel mass drug administration program for children aged 5 years and below in resource-limited settings

**DOI:** 10.1186/s13643-022-02087-z

**Published:** 2022-10-21

**Authors:** Mhlengi Vella Ncube, Muhubiri Kabuyaya, Moses John Chimbari

**Affiliations:** 1 School of Nursing and Public Health, College of Health Sciences, University of KwaZulu-Natal, Durban, South Africa; 2 Medical Affairs Institute, Johannesburg, South Africa

**Keywords:** Schistosomiasis, Treatment, Children, Praziquantel, Preschool, Patient journey map

## Abstract

**Background:**

The early childhood development of millions of children in some low- and medium-income countries may be compromised by schistosomiasis infections contracted at the age of 5 years and below. Currently, there are no standard guidelines for treating schistosomiasis in children that are 5 years and younger using praziquantel (PZQ), the only drug that the World Health Organization (WHO) recommends for treating schistosomiasis. The review is on processes and resources involved in the treatment of schistosomiasis in children aged 5 years and below.

**Methods:**

An electronic search for peer-reviewed articles published in the period from January 2011 to August 2021 was done in the Academic Search Complete, CINAHL with Full Text, Health Source: Nursing/Academic Edition, and MEDLINE databases via EBSCOHost and Google Scholar databases. The search targeted journals that described the treatment of schistosomiasis in children 5 years and below using praziquantel.

**Results:**

Thirteen studies met the inclusion criteria. The patient journey for treating schistosomiasis in children aged 5 years old and below using PZQ included the following activities: enrolment of the children into the treatment program; clinical examination; diagnosis; taking anthropometric measurements; feeding the children, making the PZQ palatable to the children; administration of PZQ; and monitoring of side effects. There was also a variation in the resources used to treat children aged 5 and below for schistosomiasis.

**Conclusions:**

A PZQ mass drug administration program for children aged 5 years old and below in endemic areas should exclude the diagnosis of schistosomiasis before treatment. The resources required in the treatment process should be affordable, and should not require skills and maintenance resources that are beyond those that are available at the primary healthcare level.

**Supplementary Information:**

The online version contains supplementary material available at 10.1186/s13643-022-02087-z.

## Background

Globally, over 240 million people mainly in impoverished communities are affected by schistosomiasis [1–3]. One-hundred and twenty-three million of the people affected by schistosomiasis are children [4], and among them, 23 million are below the age of 6 years. Ninety percent of the cases of schistosomiasis are found in sub-Saharan Africa [5, 6]. Children infected with schistosomes at the age of 5 years and below may have compromised development manifesting as stunted growth, lethargy, and cognitive and memory impairment [7]. If children infected by schistosomes at 5 years and below are not treated, their academic performance may be negatively affected, thus keeping them in the poverty cycle in the later years of their lives [7]. Treatment of schistosomiasis in children 5 years old and below is therefore important to prevent ailment, promote healthy development, and fight poverty [8].

The World Health Organization (WHO) recommends the use of praziquantel (PZQ) to treat schistosomiasis [9]. PZQ has been used to control schistosomiasis in affected communities through mass drug administration (MDA) [3]. MDA programs have excluded children aged 5 years and below based on the misconception that this age group is not exposed to schistosomiasis [10] and also because they do not attend school, which is where the programs are focused [11]. Some children in communities where schistosomiasis is endemic have been diagnosed with schistosomiasis in their first year of life [10]. These children remain infected and unwell until the age of 6 when they start school and are enrolled in school-based treatment programs. Early treatment of these children will prevent morbidity, promote early childhood development, and facilitate socio-economic development in affected communities [8].

The recommended dose of PZQ to treat schistosomiasis is 40 mg/kg [9]. Merk and Bayer, when registering PZQ, overlooked the use of the drug to treat schistosomiasis in children below the ages of 5 [12]. When the need to treat schistosomiasis in this age group was realized, the use of the drug on children was done without legal protection or evidence of safety and efficacy [12]. The WHO supported studies to determine the safety and efficacy of PZQ in children 5 years and below [12]. These studies showed that PZQ is safe and efficacious for treating schistosomiasis in children 5 years old and below [13, 14]. Hence, WHO has recommended that children aged 5 years and below in endemic areas be treated at a dose of 40 mg/kg during schistosomiasis control MDA programs [9]. However, there are no specific treatment guidelines for using PZQ in this age group.

Numerous challenges associated with treating schistosomiasis in children aged 5 years and below have been reported [12]. The PZQ tablet is bitter and difficult to swallow for young children because of its size [15]. The healthcare systems in some of the areas affected by schistosomiasis cannot afford to purchase or maintain scales [15]. In the absence of scales, WHO recommends the use of height-based dose poles to determine PZQ doses [16]. The WHO-approved dose pole does not cater to children who are less than 60 cm tall [16]. Children with stunted growth, who may need treatment, fall outside the scale of the dose pole. PZQ also causes minor side effects that require close monitoring [17]. Any treatment process that is developed needs to consider these challenges.

The research question for this review was as follows: “what are the processes and resources that are required to treat schistosomiasis in children below the age of five years?’’ We reviewed the processes followed in the use of PZQ to treat schistosomiasis in children aged 5 years and below; furthermore, the literature on the treatment of schistosomiasis in these children is limited and has not been extensively reviewed. The scoping review methodology was used to answer our research question. We reported on the PZQ treatment patient journey and resources that were used to treat schistosomiasis in children aged 5 years old and below in different clinical studies. We then developed a patient journey map and identified the resources that could be used in the implementation of a PZQ MDA program targeting children under 5 years in resource-constrained setup. MDA programs for children are essential in promoting early childhood development and contribute to ending poverty [8].

## Methods

We used the scoping review methodology for this study because scoping reviews are recommended when the purpose of the study is “to examine how research is conducted on a certain topic or field” [18]. The Arksey and O’Malley’s framework for scoping reviews with modifications from Levac et al. [19, 20] was used to describe the process and resources involved in treating schistosomiasis in children below 5 years old. However, we did not conduct the interviews outlined in Arksey and O’Malley’s framework (2010) methodology.

The searches covered a 10-year period that is January 2011 to August 2021. Only peer-reviewed journal articles reporting on primary data were included in the review. The scope of the review was limited to studies reporting on the technical activities involved in the treatment of schistosomiasis of the species *Schistosoma haematobium* and *Schistosoma mansoni* in children aged 5 and below. All studies that reported on the use of PZQ to treat schistosomiasis in children aged 5 years old and below were included. Peer-reviewed journal articles reporting on the treatment of schistosomiasis in children aged 5 years and below using drugs other than PZQ were excluded because PZQ is the only drug that has been approved by WHO for use in schistosomiasis treatment programs. Studies that reported on the use of Epiquantel were included because PZQ is an active ingredient in the Epiquantel syrup. Studies that reported on the treatment of schistosomiasis in children above 5 years were included if they also reported on the treatment of under 5 years old as a category were included.

Since it is not necessary to diagnose schistosomiasis during a PZQ MDA program [21], studies reporting exclusively on the diagnosis of schistosomiasis (and did not include treatment of children aged 5 years old and below in the methodology) were excluded. Peer-reviewed journal articles that were not written in English were also excluded because there were no translation resources.

The search terms schistosomiasis, bilharzia, treatment, praziquantel, and under 5 years old, preschool children, were used to find studies from the Google Scholar database. The following combination of search words and Boolean terms were used: schistosomiasis OR bilharzia AND treatment OR praziquantel AND children under 5 years old to search for studies from the EBSCOHost database. The EBSCOHost databases that were searched are Academic Search Complete, CINAHL with Full Text, Health Source: Nursing/Academic Edition, and MEDLINE.

The searches were initially saved on libraries created in the EBSCOHost and Google Scholar databases. Completed searches were merged onto EndNote X10. EBSCOHost removed several duplicates during the transfer process. The remaining duplicates were removed using EndNote. Title, abstract, and full article screening were used to identify articles that met our inclusion criteria.

A modified Donabedian framework was used to analyse the findings in our study [22]. The Donabedian framework separates operations into structure resources, processes, and outcomes [22]. The treatment process was described in the form of a patient journey map. We identified the processes and resources involved in the treatment of schistosomiasis in children under 5 years old. We then used the processes that were reported in the studies as a framework on which we constructed a patient journey map that can be used to implement a schistosomiasis control MDA program for children under 5 years old. We also used the resources identified in the study to select those that would be most appropriate for use in a schistosomiasis control MDA program for children aged 5 years and below in resource-limited settings.

The Preferred Reporting Items for Systematic reviews and Meta-Analyses extension for Scoping Reviews (PRISMA-ScR) checklist was used to assess the manuscript [23].

## Results

**Fig. 1:**
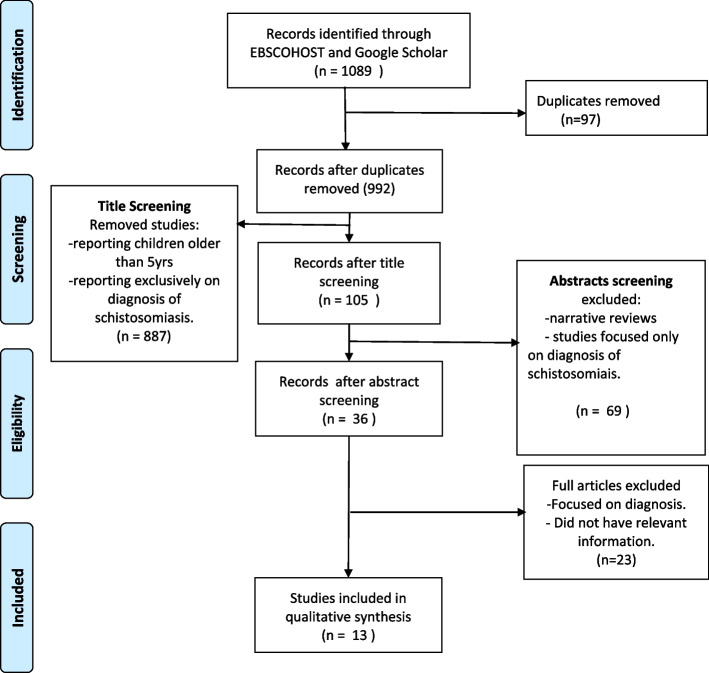
PRISMA chart describing the search process for selecting final papers for the review

Our initial search yielded 1088 titles. After removing duplicates, titles reporting exclusively on children older than 5 years, review papers, and studies that focused on diagnosis remained with 36 studies. Twenty-three of those studies were removed after full article screening because they focused on diagnosis or did not describe the processes, materials, and outcomes that were used in the treatment process. Thirteen studies met the search criteria (Fig. 1).

Thirteen peer-reviewed articles met our inclusion criteria (Table 1). The studies in these articles were from 5 different countries, namely Ghana (41.7%), Ivory Coast (16.7%), Niger (8.3%), Sudan (8.3%), and Zimbabwe (16.7%). The lowest age of the children reported in the studies was 1 month old, and the highest age reported was 93 months. Ten of the studies reported on the use of the PZQ tablet, 1 study reported on the use of PZQ syrup, and 1 study reported on the use of both the PZQ tablet and the PZQ syrup.

**Table 1:** The 12 studies that were selected for review

Author and year of publication	Country	Study title	Study aim/objective	Processes	Structure (materials)
Mutamad A. Amin, et al., 2012 [24]	Sudan	Treatment of preschool children under 6 years of age for schistosomiasis: safety, efficacy, and acceptability of praziquantel	This study investigated safety, efficacy, and acceptability of praziquantel for the treatment of *S. haematobium* and *S. mansoni* infections among preschool children aged < 6 years. The study also investigated the burden of schistosomiasis in this age group	•Enrolment of children •Clinical assessment •Diagnosis •Height and weight measurements •Treatment •Monitoring of side effects	•Registers •Questionnaires •Kato-Katz kit •Urine filtration kit •Weight scales •Stadiometer •Praziquantel •Sweetener (honey) •Food (snack)
Jean T. Coulibaly, et al., 2012 [25]	Coˆ te d’Ivoire	Efficacy and safety of praziquantel in preschool-aged Children in an area co-endemic for *Schistosoma mansoni* and *S. haematobium*	The study reported here was designed to assess the efficacy and safety of crushed praziquantel tablets in preschool-aged children (6 years) in an area where *Schistosoma manson*i and *S. haematobium* coexist	•Enrolment of children •Clinical examination •Laboratory diagnosis •Weight measurements •Treatment •Monitoring of side effects	•Registers •Questionnaires (side effects only) •Kato-Katz kit •Schistosomiasis urine filtration kit •POC-CCA cassettes •Weight scales •PZQ •Spoons •Water
Jean T. Coulibaly, et al., 2017 [26]	Coˆ te d’Ivoire	Efficacy and safety of praziquantel in preschool-aged and school-aged children infected with *Schistosoma mansoni*: a randomized controlled, parallel-group, dose-ranging, phase 2 trial	To determine the nature of the dose response of praziquantel in PSAC infected with *S mansoni* and to determine the dose of praziquantel that shows an efficacy comparable to the standard dose of 40 mg/kg in SAC in an area where *S mansoni* is endemic	•Enrolment •Clinical examination •Diagnosis •Weight measurements •Treatment •Monitoring of side effects	•Registers •Questionnaires •Kato-Katz kit •Urine filtration kit •POC-CCA cassette •PZQ •Food (bread) •Sweetener (syrup-flavoured water) •Pestle and mortar
Jean T. Coulibaly, et al., 2018 [27, 28]	Côte d’Ivoire	Efficacy and safety of ascending doses of praziquantel against *Schistosoma haematobium* infection in preschool-aged and school-aged children: a single-blind randomised controlled trial	To assess the efficacy and safety of escalating praziquantel dosages in PSAC infected with *Schistosoma haematobium*	•Enrolment •Clinical assessment •Diagnosis •Weight measurements •Treatment •Monitoring of side effects	•Registers •Questionnaires •Schistosomiasis urine filtration kit •Food •Weight scales •PZQ •Pestle and mortar •Sweetener (syrup-flavoured water) •Kato-Katz kit
Amadou Garba, et al., 2013 [14]	Niger	Safety and efficacy of praziquantel syrup (Epiquantel®) against *Schistosoma haematobium* and *Schistosoma mansoni* in preschool-aged children in Niger	The objective of the current study was to assess the safety and efficacy of Epiquantel® in preschool-aged children in Niger in order to provide additional evidence for the treatment of this age group with praziquantel as part of the ongoing preventive chemotherapy control programs implemented in highly endemic areas	•Enrolment •Clinical examination •Diagnosis •Weight measurements •Treatment •Monitoring of side effects	•Questionnaire •Child Health Booklet •Urine filtration kit •Kato-Katz kit •Weight scales •Food (millet wafer and porridge) •PZQ syrup •Millimetre scale pipette •Bottled water •Cup
Francisca Mutapi, et al., 2011 [29]	Zimbabwe	*Schistosoma haematobium* treatment in 1–5-year-old children: safety and efficacy of the antihelminthic drug praziquantel	This study investigated the safety and efficacy of PZQ treatment in such children	•Enrolment •Clinical examination •Diagnosis •Weight measurements •Treatment •Monitoring of side effects	•Registers •Questionnaire •Kato-Katz kit •Schistosomiasis urine filtration kit •Weight scales •PZQ •Sweetener (juice) •Spoons •Food (bread)
Allen Nalugwa, et al., 2015 [30]	Uganda	Single- versus double-dose praziquantel comparison on efficacy and *Schistosoma mansoni* reinfection in preschool-age children in Uganda: a randomized controlled trial	Designed to compare the efficacy of PZQ in terms of CRs and ERRs using single- and double-dose regimens and its effect on *S. manson*i reinfection 8 months post treatment in children aged 1–5 years living along Lake Victoria in eastern Uganda	•Enrolment •Diagnosis •Weight measurement •Treatment •Monitoring of side effects	•Register •Questionnaire (side effects) •Kato-Katz kits •Weight scales •PZQ •Sweetener (orange juice) •Water •Food (bread and orange juice)
Harriet Namwanje et al., 2011 [31]	Uganda	The acceptability and safety of praziquantel alone and in combination with mebendazole in the treatment of *Schistosoma mansoni* and soil-transmitted helminthiasis in children aged 1–4 years in Uganda	To determine the acceptability and safety of praziquantel alone and in combination with mebendazole in the treatment of *S. mansoni* and STH in children aged 1 to 4 years	•Enrolment •Clinical examination •Diagnosis •Weight measurements •Treatment •Monitoring of side effects	•Registers •Questionnaires •Specimen containers •Kato-Katz kits •Weight scales •PZQ •Mebendazole
A. M. D. Navaratnam, et al., 2012 [13]	Uganda	Efficacy of praziquantel syrup versus crushed praziquantel tablets in the treatment of intestinal schistosomiasis in Ugandan preschool children, with observation on compliance and safety	In this study, the performance of Epiquantel in terms of therapeutic efficiency, noncompliance, and side effects was assessed under field conditions alongside the crushed tablet alternative	•Enrolment •Clinical examination •Diagnosis •Weight measurements •Treatment •Monitoring of side effects	•Registers Questionnaire (side effects) Kato-Katz kits •Weight scales •PZQ •PZQ syrup •Paracetamol •Sweetener (juice) •Food (bread)
Jose´ C. Sousa-Figueiredo, et al., 2012 [16]	Uganda	Performance and safety of praziquantel for treatment of intestinal schistosomiasis in infants and preschool children	This study therefore aimed to assess the performance and safety of PZQ treatment in under 7 year olds living in *Schistosoma mansoni*-endemic areas	•Enrolment •Clinical examination •Diagnosis •Weight measurements •Treatment •Monitoring of side effects	•Registers •Questionnaires •Kato-Katz kit •Weight scales •PZQ •Sweetener (orange juice) •Food (bread) •Spoons
José Carlos Sousa-Figueiredo et al., 2010 [32]	Uganda	Treatment of intestinal schistosomiasis in Ugandan preschool children: best diagnosis, treatment efficacy and side effects, and an extended praziquantel dosing pole	To provide detailed evidence on occurrence of intestinal schistosomiasis in very young children (≤ 6 years of age), to observe the efficacy and safety of PZQ in this age class and extend the current dose pole to facilitate the allocation of treatments within mass drug administration initiatives	•Enrolment •Clinical examination •Diagnosis •Weight and height measurements •Treatment •Monitoring of side effects	•Registers •Questionnaires •Kato-Katz kit •SEA-ELISA •POC-CCA •Weight scales •PZQ •Sweetener (orange juice and sugar) •Stadiometer
Welcome M. Wami et al., 2016 [33]	Zimbabwe	Comparative assessment of health benefits of praziquantel treatment of urogenital schistosomiasis in preschool and primary school-aged children	To determine the effect of single praziquantel treatment on *Schistosoma haematobium*-related morbidity markers: microhaematuria, proteinuria, and albuminuria	•Enrolment •Clinical examination •Diagnosis •Weight measurements •Treatment •Monitoring of side effects	•Registers •Questionnaires •Schistosomiasis urine filtration kit •Kato-Katz kit •Urinalysis dipsticks •CLINITEK Status + Analyzer •CLINITEK Microalbumin Reagent Strip •Weight scales •PZQ •Sweetener (juice) •Food (bread)
Makida Kemal, 2019 [66]	Ethiopia	*Schistosoma mansoni* infection among preschool age children attending Erer Health Center, Ethiopia, and the response rate to praziquantel	To assess *S. mansoni* infection in PSAC and the response rate for praziquantel treatment (40 mg/kg)	•Enrolment •Clinical examination •Laboratory diagnosis •Treatment •Monitoring of side effects	•Registers •Questionnaires •Kato-Katz kit •Weight scales •PZQ

### Outcomes

All the studies that we reviewed reported that PZQ is efficacious against schistosomiasis in children under 5 years. The lowest PZQ dose that was efficacious against schistosomiasis in this age group was 20 mg/kg [26, 27]. All the studies reported on the PZQ dose of 40 mg/kg to treat schistosomiasis in this age group. Three studies reported the difference in PZQ efficacy observed between the use of a PZQ dose of 40 mg/kg and that of 60 mg/kg in the treatment of schistosomiasis in children aged 5 years old and below [26, 28, 29].

All the studies reviewed stated that PZQ is safe for use in the treatment of schistosomiasis in children aged 5 years old and below. The highest safe dosage of PZQ reported was 60 mg/kg [26, 27]. The difference in PZQ efficacy observed between the use of a PZQ dose of 40 mg/kg and that of 60 mg/kg in the treatment of schistosomiasis in children aged 5 years old and below was insignificant [26, 28, 29]. All the studies reported that there were no serious adverse events reported after treating schistosomiasis in children aged 5 years and below with PZQ doses of between 20 to 60 mg/kg. The WHO-recommended dose of 40 mg/kg should be used to treat schistosomiasis during schistosomiasis control MDA programs for children aged 5 years old and below.

### Process

All the studies followed the same treatment sequence: enrolment of children into the program, clinical examination, diagnosis, weight and height measurements, and treatment and the monitoring of side effects. Enrolment involved, acquiring consent from parents, recording and clinically examining all the children that participated in the study to make sure that they were safe to receive PZQ treatment. Children who were considered safe for treatment were those who were generally well [13, 14, 24, 25, 33, 34], had no recent illness [33], were not suffering from or receiving treatment for tuberculosis (TB) [33], did not have malaria [13], had not undergone a major surgical procedure [33], were not suffering from a fever [31, 33], and did not have a history of adverse drug reactions [31]. The appropriate interventions were provided to the enrolled children who were ineligible for PZQ treatment based on ill health.

Diagnosis of schistosomiasis was done in all the studies to determine baseline prevalence and intensity in the children before treatment. Weight and height [16, 26, 32] measurements were then done to determine the required dose of PZQ to treat children. Administration of PZQ included feeding the children to reduce side effects [30] and to increase the assimilation of PZQ [14, 34], making the PZQ palatable for the children by breaking or crushing the tablet and mixing it with a sweeter and feeding the tablets to the children. After orally taking the tablet, the children were monitored for side effects. The most immediate side effect that was observed was vomiting the tablet within the first 20–60 min [25, 29], in which case the tablet was re-administered to the children. Other side effects were recorded, and appropriate treatment was provided to the children.

**Fig. 2:**
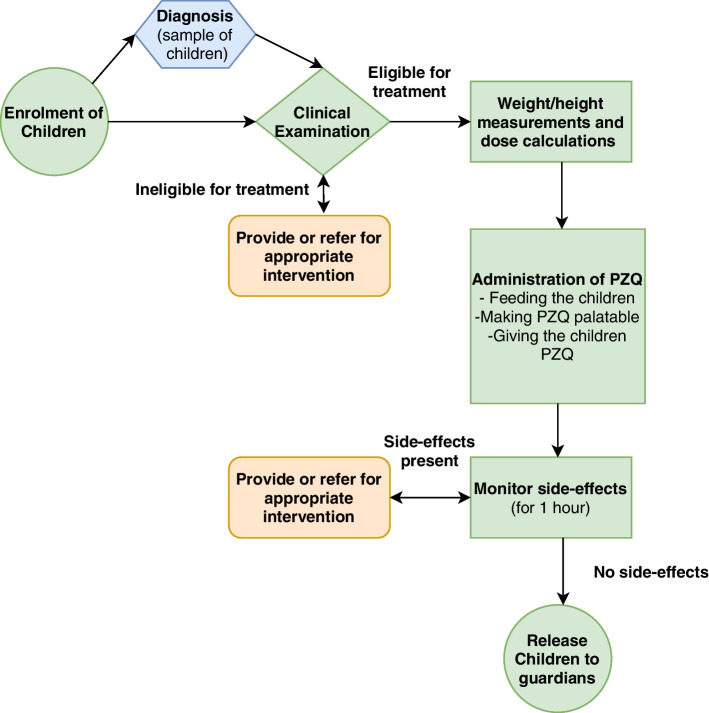
Patient journey map for a schistosomiasis control MDA program for children aged 5 years old and below

Using the processes carried out in the 13 clinical studies to treat schistosomiasis in children aged 5 years and below, we mapped a patient journey that could be used to implement a schistosomiasis control MDA program for this age group (Fig. 2). In the PZQ MDA patient journey, diagnosis of schistosomiasis could be limited to a sample of the children before for surveillance purposes. The collection of biological specimens for diagnosis could be done before the clinical examination of the children.

### Structure

The materials that were used to treat schistosomiasis in children under 5 years old in the studies are listed in Table 2. In the same table, we have proposed materials that could be used in a schistosomiasis MDA program for children under 5 years old in resource-limited settings. Registers were used to enroll children in the clinical studies. For a schistosomiasis control MDA program for children under 5, we recommend electronic registers that are embedded in electronic tablets. The Kato-Katz and schistosomiasis urine filtration kits [24–27, 29, 30, 33, 35] were the most used materials to diagnose *Schistosomiasis mansoni* and *Schistosomiasis haematobium*, respectively. The stools and urine for diagnosis were collected in specimen containers [31]. In serological tests, point-of-care circulating cathodic antigen (POC-CCA) [25, 26, 32], soluble egg antigen enzyme-linked immunosorbent assay SEA-ELISA [32], microalbumin reagent strips [33], and urinalysis dipsticks [33] were also used to test for schistosomiasis in the children. Questionnaires were used for diagnosis [4, 14, 16, 24, 26, 27, 29, 32, 33]. We recommend the use of questionnaires and urine dipsticks for the diagnosis of urinary schistosomiasis and the POC-CCA for the diagnosis of intestinal schistosomiasis for disease surveillance in schistosomiasis control MDA programs for children aged 5 years and below. Questionnaires and the Child Health Booklet [14] were used for clinical assessments. We also recommend that both be used for clinical assessments in the schistosomiasis control MDA programs for children aged 5 years and below. Some of the studies that we reviewed reported using weight scales, while others used stadiometers [24, 32] and one used dose poles [32] to obtain the anthropometric measurement that were used to calculate the dose amount of PZQ. We recommend the use of the dose pole and/or tape measures to make these measurements in schistosomiasis control MDA programs for young children.

**Table 2:** Materials required in the treatment of schistosomiasis in children aged 5 years old and below

Activity	Structure(materials and human resources) reported	Structure (materials) recommend for MDA in resource-limited settings		Reasons for selection
Enrolment	Registers		Tablets	Eco-friendly Easy to use
Diagnosis	CLINITEK Status + Analyzer ClINITEK Microalbumin Reagent Strip Kato-Katz kit POC-CCA kit Questionnaires SEA-ELISA Schistosomiasis urine filtration kit Specimen containers Urinalysis dip sticks		Urine dipsticks POC-CCA	Easy to use Easy to use
Clinical examination	Questionnaires Child Health Booklet		Child Health Booklet Questionnaire	Easy to access Easy to use
Weight/height measurements	Dose pole Stadiometers Weight scale		Dose pole Tape measures	Low cost Easy to maintain
Food and sweetener	Bread Honey Juice Millet wafer Porridge Syrup-flavoured water Sugar		Bread Juice	Easy to access Easy to access
Administration of PZQ	Cups Millimetre pipette Pestle and mortar PZQ syrup PZQ tablets Spoons Water		Pestle and mortar Spoons PZQ tablets	Low cost Low cost and easy to use
Infrastructure	Clinics ECD centres Faith-based establishments Healthcare centres Schools		Clinics Crèches/ECD centres Faith-based establishments	Easy to access Easy to access Already used for some healthcare programs
Human resources	Community health workers Laboratory technicians Medical doctors Nurses Paediatricians		Community health workers Nurses	Available in situ Available in situ

Bread and juice [13, 26, 29, 30, 33, 34], millet wafer [14], and porridge [14] were the food items that were used in the studies we reviewed. PZQ tablets were crushed with spoons [29] or pestle and mortar [26, 27] to make them small enough for the children to take in. The studies used honey and sugar [24], juice, and syrup flavoured water as sweeteners [13, 26, 29, 30, 33, 34] to mask the bitter taste of PZQ. We recommend that children aged 5 years old and below should be fed with bread and juice or instant porridge during schistosomiasis control MDA programs for this age group. We recommend the use of tablets that are crushed with a pestle and mortar, with juice as the sweetener for use in these MDA programs.

Some of the studies reported using schools [29, 31, 33], early childhood development (ECD) centers [33], healthcare centres [13], clinics [32], and churches [13] as sites where the children were recruited. The schools were used as treatment centres in studies that involved comparisons between children that were aged 5 years old and below (of preschool aged children (PSAC)) and school-aged children (SAC) [33]. The ECD centres that are reported on in the studies were attached to the schools where the SAC participants were found [33]. We recommend the use of schools and clinics as treatment centres for schistosomiasis control MDA programs for children aged 5 years old and below.

The human resources that took part in the clinical aspects of the studies were from a variety of healthcare professions. These medical staff included pediatricians [31], medical doctors [14], nurses [29], and laboratory technicians [26, 27]. Some studies did not specify the professions of the clinical human resources only indicating that medical staff, clinicians, or health officers were part of the study. One study stated the involvement of community leaders and community drug distributors [32] as part of their human resources. We recommend nurses to be the healthcare professionals that implement the schistosomiasis control MDA programs for children aged 5 years old and below.

### Challenges

Some of the studies reported the use of the current WHO-approved 600 mg PZQ dose tablet formulation as a challenge when treating schistosomiasis in children aged 5 years old and below, especially when implementing a large-scale treatment program [26, 30, 36]. They recommended that a variation of this formulation with three partitions that make it possible to split the tablet into four pieces of 150 mg each should be used when treating schistosomiasis in children aged 5 years old and below to ensure correct usage of PZQ to treat schistosomiasis in these children [32]. The studies proposed that a pediatric formulation should be developed, and that the PZQ pediatric formulations could be an orally dispensable tablet [26] or a PZQ syrup [14].

## Discussion

The objective of this study was to map processes and resources that are required to implement a schistosomiasis control MDA program for children aged 5 years old and below. The implementation of a schistosomiasis control MDA program for children aged 5 years old and below has not been reported. The closest to a schistosomiasis MDA program for this age group is mainly clinical studies that were recommended by WHO to determine the safety and efficacy of treating schistosomiasis in children aged 5 years old and below using PZQ [12]. We modified the processes that were used in these studies to propose a treatment process or patient journey map that could be used to implement a schistosomiasis control MDA program for children aged 5 years and below.

### Process

The patient journey for a PZQ MDA program for children aged 5 years and below could follow the sequence: enrolment of children, clinical examination, weight and height measurements, treatment, and the monitoring of side effects for all the children. This is in line with recommendations of the WHO that individual diagnosis of schistosomiasis before treatment is not required before treatment during an MDA program [21]. The studies we reviewed diagnosed all the children to determine the prevalence of schistosomiasis in the children that are aged 5 years and below and also to determine the efficacy of PZQ in treating schistosomiasis in these children. The diagnosis of schistosomiasis in the MDA program is important to determine the baseline prevalence and burden of infectivity of schistosomiasis in the children for monitoring and evaluation [37]. Diagnosis of schistosomiasis in a schistosomiasis control MDA program for children aged 5 years and below for monitoring and evaluation could be done on a random sample of the children soon after they have been enrolled into the treatment program [38]. In some cases, the baseline prevalence and infection intensity could be done separately from the MDA program during disease surveillance programs making the treatment process to move directly from enrolment of the children to clinical assessments [39]. The disease surveillance programs when present could be used to monitor and evaluate the schistosomiasis control MDA programs for children aged 5 years and below.

### Structure

The Donabedian framework refers to resources as structural components of a healthcare system [22]. The resources that are required to implement a schistosomiasis control MDA program for children aged 5 years and below should be field applicable to the settings in which the MDA program will be implemented [40]. Most of the places where schistosomiasis is endemic are economically disadvantaged and therefore suffer from resource constraints. We used relative cost and ease of access to select the resources that we recommended to be the best for use in a schistosomiasis control MDA program for children aged 5 years and below. The resources used should also be eco-friendly [41].

None of the studies described the nature of the registers that they used for enrolment. Electronic registers that are embedded in tablets could be used to enrol children into a schistosomiasis treatment programs. The use of electronic information management systems in the MDA program could be extended to storing and analysing the clinical assessments of the children to identify other childhood illnesses that need mass intervention. Tablets can be used for controlling the quality of the medical information about the children that is requested using questionnaires, for managing and monitoring the program’s consumables inventory, and for monitoring and evaluation of the progress and impact of the MDA program. A multinational study conducted in low- and medium-income countries (Ghana, Kenya, India, and Pakistan) reported that the initial cost of purchasing electronic tablets and software licensing costs for healthcare program data management could be high [42]. However, because the tablets could be used multiple times for the same program and also across different programs, the purchase of tablets could be a justifiable investment when taking into account economies of scale and economies of scope in healthcare program implementation cost analysis [42, 43]. The limitations of using electronic tablets could include intermittent electricity supply and Internet coverage in some of the areas where the schistosomiasis control MDA program could be necessary [42, 44]. The use of paper-based registers is most common and carries some hidden costs [42]. When the initial cost of buying and setting up electronic systems for treatment data management is unaffordable, paper-based registers could be used.

The WHO recommends that diagnosis should not be made a requirement for the mass drug administration of praziquantel in schistosomiasis-endemic areas [21]. Diagnosis could however be used for monitoring and evaluation of a schistosomiasis control MDA program. To diagnose schistosomiasis in children aged 5 years old and below for monitoring and evaluating a schistosomiasis control MDA program targeting this age group, urine dipstick to detect haematuria caused by urinary schistosomiasis and the POC-CCA to detect intestinal schistosomiasis could be used. Both these methods have the advantages of being performed at the site of the MDA program and are more sensitive than the microscopy-based methods that are normally used to diagnose schistosomiasis in school-going children [40, 45, 46]. The urinary dipstick and POC-CCA point-of-care tests do not require specialized human resources, in particular laboratory technicians, to carry out the testing. Other methods such as the polymerase chain reaction (PCR) and the FLOTAC technologies require additional specially skilled technicians and equipment such as thermocyclers and centrifuges which cannot be used in field [47].

The activities that are involved in treating schistosomiasis in children aged 5 years and below include taking weight or height measurements, calculating dosage, feeding the children, making PZQ more palatable to the children, and administering the PZQ. Height rather than weight could be used to determine the dose of PZQ to be given to the children [16]. This is because the dose poles that are used to measure height are less expensive to buy and maintain compared to the weight scales that could be used if weight is used to determine the dosage [48]. The extended dose pole addresses the limitation of the exclusion of children shorter than 94 cm from being treated based on the dose pole [16]. The use of electronic health systems management could eliminate the need for dose poles and weight scales by providing data that could be used to perform weight-for-age calculations [49]. The age of the child could then be used to calculate the appropriate PZQ dose to treat the children [49].

We recommend that bread and juice are the most suitable foods to feed the children before treatment. This is because bread and juice are filling and yet easily available in most communities and also require very little labour to prepare [50]. Fortified spreads could be used on the bread to enhance its nutritional value [51]. Juice also has the added capability to sweeten the PZQ making it more palatable for the children [52]. An alternative to bread and juice could be fortified cereal [53]. Fortified cereal has the advantage of providing multi-nutrients to children [53], thereby addressing the needs of the schistosomiasis control MDA program for children aged 5 years old and below, tackling the challenge of malnutrition [54] especially since schistosomiasis and childhood malnutrition often exist as co-epidemics [15, 54]. The studies reported using either pestle and mortar or spoons to crush the PZQ tablets. Ideally, the pestle and mortar could be used. This is because the pestle and mortar are the best tools to use to crush the tablets and are also inexpensive [55]. Spoons are less efficient in crushing tablets but could also be used when pestle and mortar are not available.

Schistosomiasis control MDA programs for children aged 5 years old and below could be carried out in clinics or schools. The use of clinics for such an MDA program is encouraged by the WHO [9]. Schools on the other hand have been successfully used by schistosomiasis control MDA programs to treat SAC [29, 56]. Since some ECD centres are attached to schools, the same operational strategies that have been used to target SAC could be used to target pre-school-going children (PSAC) [29, 33]. Children who are not enrolled in ECD centres could be invited to be part of MDA programs. Faith-based establishments such as churches and mosques are used for healthcare programs in some resource-limited settings [57, 58] and could be used to mobilize people to enrol their children who are aged 5 years and below into schistosomiasis control MDA programs [59, 60]. Faith-based establishments could also be used as treatment sites for the MDA program. The choice between using clinics, ECD centres, crèches, and faith-based establishments depends on the prevailing government policy and on where the children under 5 years who are at risk of schistosomiasis could be best accessed for treatment in the community where the MDA program is required.

The studies we reviewed reported on using a variety of human resources such as paediatricians, medical doctors, nurses, laboratory technicians, and community health workers. Paediatricians, medical doctors, and laboratory technicians are seldom available in resource-limited settings such as those that are affected by schistosomiasis [61]. Nurses could therefore implement the schistosomiasis control MDA program for children aged 5 years old and below. This is because nurses have been found to successfully implement paediatric healthcare interventions at the primary healthcare level in the resource-limited setting where schistosomiasis is mostly found [62]. Community health workers are needed to support the clinicians in MDA programs because of human resources constraints in most areas that are affected by schistosomiasis [63, 64]. Considerations of the disruption of routine curative functions of the nurses in the clinics where the nurses normally work and the remuneration motivation of community caregivers should be taken into account when developing a human resources strategy for the implementation of an MDA program [61, 65] to control schistosomiasis in children aged 5 years and below.

The strength of this study is that the studies are from 5 different countries which makes them generalizable to resource-limited settings in Africa and similar settings globally. The limitations of the study are that we did not perform interviews as recommended by Arksey and O’Malley’s framework for systematic reviews [19]. The interviews could have given us more information on the resources that are applicable for use in schistosomiasis control MDA programs for children under 5 years in different settings. We, however, could not perform these interviews due to challenges in getting authorization from several countries to conduct the interviews within the duration of the study. Another limitation of the review was that the inclusion criterion excluded studies that were not in English because there were no interpreters. The exclusion of studies that were written in other languages besides English could have created bias. The diversity of countries in which studies were done alleviates the impact of the language bias.

## Conclusion

The patient journey that could be followed in the implementation of a schistosomiasis control MDA program for children aged 5 years old and below is enrolment of children into the treatment program, clinical examination, weight and height measurements, treatment, and the monitoring of side effects for all the children (Fig. 2). Diagnosis could be done for monitoring and evaluation either before the MDA program or immediately after the enrolment stage of the MDA program. The resources that could be used for the treatment program are as follows: electronic tablets, urine dipsticks, POC-CCA, child health booklet, questionnaire, dose pole, tape measures, bread, juice, pestle and mortar, spoons, PZQ tablets, clinics, and ECD centres. The availability of these resources in different healthcare centres may vary resulting in the need for unavailable resources to be purchased before the program. Resources that are already available and routinely in use at the primary healthcare care level should be prioritized for use in the MDA program.

## Supplementary Information

**Additional file 1.** Preferred Reporting Itemsfor Systematic reviews and Meta-Analyses extension for Scoping Reviews(PRISMA-ScR) Checklist.

## Data Availability

Data will be made available upon request.

## Abbreviations

ALB: Albendazole
ECD: Early childhood development
HAART: Highly active antiretroviral therapy
MDA: Mass drug administration
MEDLINE: Medical Literature Analysis and Retrieval System Online
POC-CCA: Point-of-care circulating cathodic antigen
PSAC: Preschool aged children
PZQ: Praziquantel
SAC: School-aged children
SEA-ELISA: Soluble egg antigen enzyme-linked immunosorbent assay
STH: Soil-transmitted helminths
TB: Tuberculosis
UKZN: University of KwaZulu-Natal
WHO: World Health Organisation
